# A Rare Case of Secondary Postpartum Hemorrhage Due to Intrauterine Hematoma and Uterine Scar Dehiscence

**DOI:** 10.7759/cureus.107703

**Published:** 2026-04-25

**Authors:** Parth Dhande, Kanchan S Dwidmuthe, Anuja Bhalerao, Savita Somalwar

**Affiliations:** 1 Department of Obstetrics and Gynaecology, NKP Salve Institute of Medical Sciences and Research Centre, Lata Mangeshkar Hospital, Nagpur, IND; 2 Department of Obstetrics and Gynaecology, NKP Salve Institute of Medical Sciences and Research Centre, Nagpur, IND

**Keywords:** cesarean, intrauterine hematoma, postpartum hemorrhage, scar dehiscence, secondary postpartum hemorrhage, uterine scar dehiscence

## Abstract

Cesarean section is one of the most frequently performed obstetric procedures worldwide. Although secondary postpartum hemorrhage (PPH) after cesarean section is uncommon, it remains a potentially life-threatening condition and a significant cause of maternal morbidity, even in regions with low maternal mortality. Secondary PPH typically occurs between 24 hours and 12 weeks postpartum. Uterine scar dehiscence is a rare but important cause of secondary PPH, often posing diagnostic and therapeutic challenges. We present a case of a 29-year-old female who developed secondary PPH 60 days after an uncomplicated lower-segment cesarean section.

## Introduction

Secondary postpartum hemorrhage (PPH) is defined as abnormal or excessive vaginal bleeding occurring between 24 hours and 12 weeks after childbirth [1]. Although many clinicians consider a six-week postpartum period, broader definitions extend up to 12 weeks, and presentations beyond six weeks are relatively uncommon and underreported. The incidence of secondary PPH ranges from 0.2% to 0.8% and represents one of the leading causes of hospital readmission in the postpartum period [2].

Common etiologies include endometritis, retained products of conception, subinvolution of the placental site, and complicated vagino-perineal trauma. Rare causes include pseudoaneurysm of the uterine artery, arteriovenous malformations, and partial or complete dehiscence of the lower uterine segment incision [3]. Although cesarean section is one of the most frequently performed obstetric procedures, its postoperative course may occasionally be complicated by uterine scar dehiscence. Secondary PPH following cesarean section occurs in approximately one in 365 cases [4].

Uterine scar dehiscence is characterized by the partial separation of a previous cesarean scar, which may lead to severe bleeding, anemia, or hemodynamic shock [5]. In severe cases, patients may present with signs of peritonitis. Therefore, prompt diagnosis and appropriate management are essential. Depending on the severity and extent of bleeding, management options include pharmacological measures, mechanical interventions, or surgical procedures such as laparotomy and hysterectomy [6].

We present a case of secondary PPH due to intrauterine hematoma and cesarean scar dehiscence, which was initially managed conservatively but ultimately required subtotal obstetric hysterectomy. This case is reported due to its unusually delayed presentation at 60 days postpartum, absence of identifiable infection, and association with intrauterine hematoma leading to uterine scar dehiscence, highlighting diagnostic and management challenges.

This case is notable for its delayed presentation at 60 days postpartum and absence of an identifiable infectious etiology.

## Case presentation

A 29-year-old female (para 3, living 2) with a history of intrauterine device (IUD) use presented to our institute 60 days after a lower-segment cesarean section (LSCS) with complaints of abdominal pain and excessive vaginal bleeding for the preceding two to three days. The bleeding was moderate to heavy, requiring four to five pads per day, and not associated with passage of clots, fever, chills, or rigors. She had no history suggestive of diabetes mellitus, asthma, or thyroid disorders. There was no significant past medical or surgical history.

The previous LSCS had been performed at another center for a term pregnancy, delivering a live male neonate weighing 2.5 kg. The postoperative course was reportedly uneventful, and she was discharged on the fifth postoperative day.

On examination, the patient was conscious, oriented, and moderately pale. Her general condition was fair, afebrile, with a pulse rate of 72 beats/min and blood pressure (BP) of 110/70 mmHg. Cardiovascular and respiratory system examinations were within normal limits. Abdominal examination revealed mild distension without guarding, rigidity, or tenderness.

Initial laboratory reports showed severe anemia (hemoglobin: 6.5 g/dL; hematocrit: 20.1%; RBC: 2.44 m/µL) and leukopenia (total leukocyte count: 2.5 ×10³/µL). Liver and renal function tests were within normal limits. Blood and urine cultures were sterile (Table 1).

**Table 1:** Baseline laboratory investigations. SGOT: serum glutamic oxaloacetic transaminase; SGPT: serum glutamic pyruvic transaminase; AST: aspartate aminotransferase; ALT: alanine aminotransferase; β-hCG: β-human chorionic gonadotropin.

Category	Investigation	Result	Normal range
Complete blood count (CBC)	Hemoglobin (Hb)	6.5 g/dL	12-16 g/dL
	Total leukocyte count (TLC)	2.5 ×10³/µL	4-11 ×10³/µL
	Platelet count	87,000 /µL	150,000-450,000 /µL
	Red blood cell count	2.44m/µL	4.1-5.1m/ µL
Kidney function test (KFT)	Blood urea	10 mg/dL	7-20 mg/dL
	Serum creatinine	0.45 mg/dL	0.6-1.2 mg/dL
	Uric acid	2.7 mg/dL	2.4-6.0 mg/dL
Liver function test (LFT)	SGOT (AST)	32 U/L	5-40 U/L
	SGPT (ALT)	45 U/L	7-56 U/L
	Alkaline phosphatase (ALP)	101 U/L	44-147 U/L
	Total bilirubin	0.5 mg/dL	0.2-1.2 mg/dL
	Total protein	7.5 g/dL	6-8 g/dL
	A/G ratio	1.34	1.0-2.2
Serum electrolytes	Sodium (Na⁺)	139 mEq/L	135-145 mEq/L
	Potassium (K⁺)	4.7 mEq/L	3.5-5.0 mEq/L
	Chloride (Cl⁻)	104 mEq/L	98-106 mEq/L
Blood glucose	Random blood sugar (RBS)	134 mg/dL	70-140 mg/dL
Coagulation profile	International normalized ratio (INR)	1.17	0.8-1.2
	Activated partial thromboplastin time (aPTT)	32.2 sec	25-35 sec
Thyroid function test	Thyroid-stimulating hormone (TSH)	2.23 µIU/mL	0.4-4.0 µIU/mL
	Triiodothyronine (T3)	5.67 pmol/L	3.1-6.8 pmol/L
	Thyroxine (T4)	17.98 pmol/L	12-22 pmol/L
Hormonal test	β-hCG	>2 mIU/mL	<5 mIU/mL (non-pregnant)

The presence of leukopenia and thrombocytopenia raised suspicion of underlying infection, consumptive coagulopathy, or bone marrow suppression. However, the absence of fever, negative blood and urine cultures, and normal coagulation parameters made overt sepsis or disseminated intravascular coagulation less likely. These abnormalities were considered possibly secondary to ongoing blood loss, hemodilution, or early consumptive processes.

Ultrasonography of the abdomen and pelvis revealed a bulky uterus (12.4 × 6.4 × 5.4 cm) with a completely echogenic endometrial cavity. Hypoechoic areas with moving internal echoes were noted in the fundal region, suggestive of an intrauterine hematoma with active bleeding. Bilateral ovaries and adnexa appeared normal.

Within five hours of admission, the patient developed hypertension (BP: 140/90 mmHg) with minimal pericardial effusion on 2D echocardiography. Pallor had worsened to a moderate-to-severe grade.

Empirical broad-spectrum antibiotics (cefuroxime 1.5 g IV, gentamicin 80 mg IV, and metronidazole 500 mg IV) were initiated considering the possibility of occult uterine infection, which is a known cause of secondary PPH, despite the absence of overt clinical or microbiological evidence. Supportive therapy included analgesics, antiemetics, and anti-fibrinolytic therapy (tranexamic acid 1 g IV).

Considering these findings, a diagnosis of severe secondary PPH was established. The clinical team decided to proceed with an emergency subtotal obstetric hysterectomy to control the ongoing hemorrhage and identify the underlying cause. High-risk informed consent was obtained from the family.

Under all aseptic precautions, the abdomen was opened through a low transverse (Pfannenstiel) incision. The uterus was flabby. Bilateral uterine artery ligation was performed, after which uterine tone was achieved. However, hemostasis was still not satisfactory, and active bleeding persisted.

**Figure 1:**
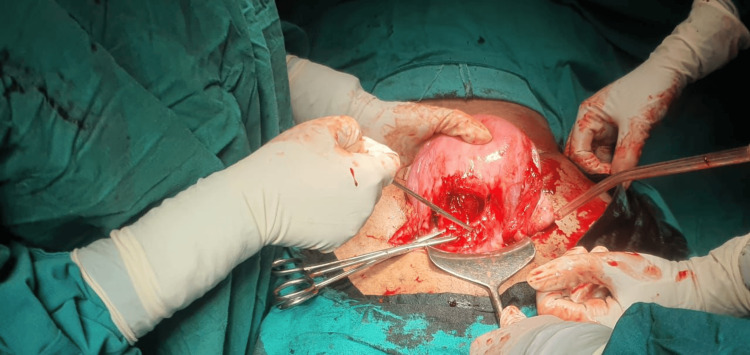
Intraoperative image demonstrating complete cesarean scar dehiscence with active bleeding.

On entering the peritoneal cavity, a hemorrhagic collection with frank blood was noted, correlating with preoperative ultrasonographic findings of an intrauterine hematoma. Intraoperative examination revealed a complete dehiscence of the cesarean scar (Figure 1).

**Figure 2:**
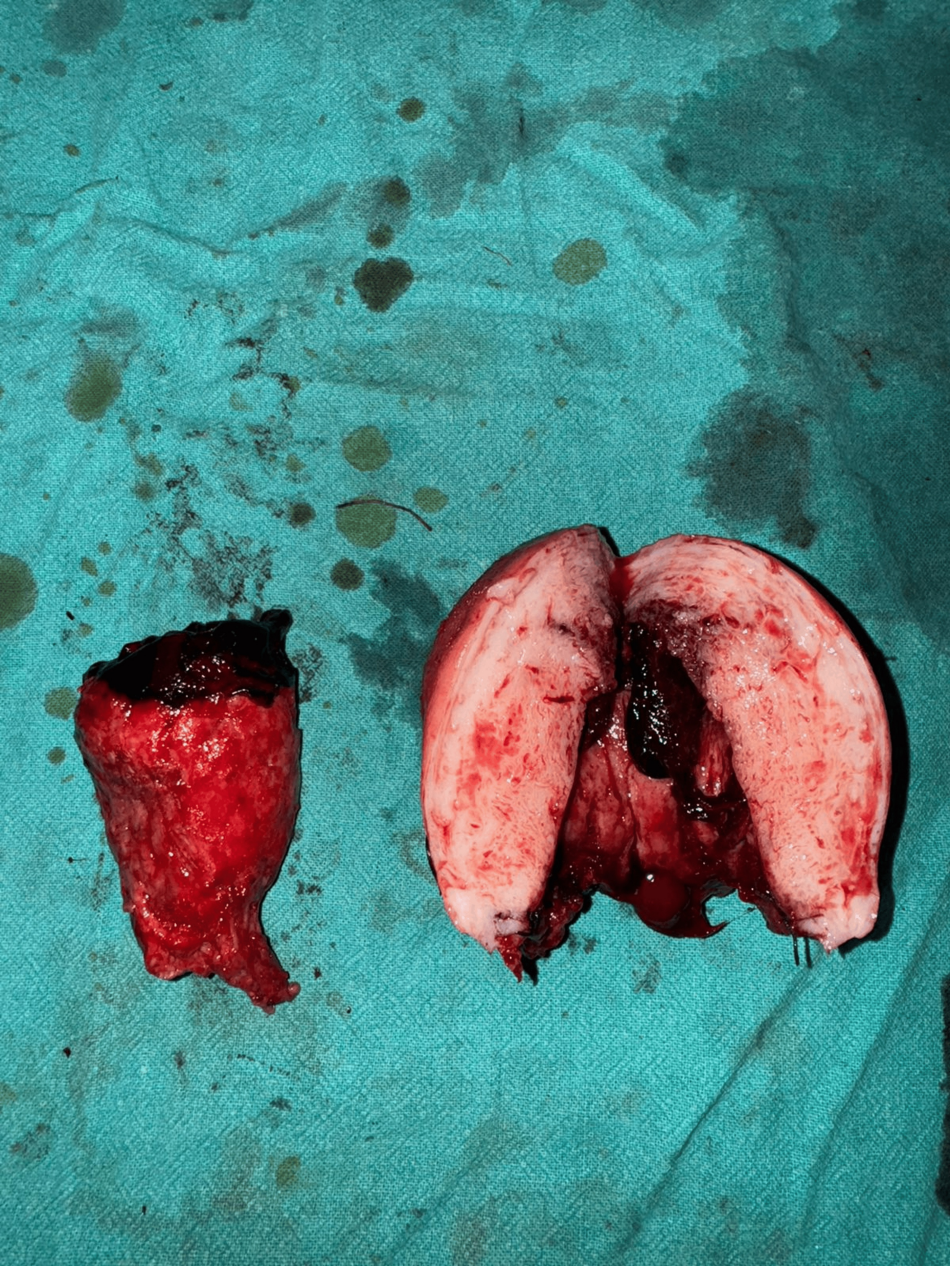
Hysterectomy specimen showing uterus with cast, likely representing organized clot or decidual tissue.

Suspicious tissue at the scar site (possibly decidual cast, placental polyp, or endometrial cast) was excised and sent for histopathological examination (Figure 2).

Despite ongoing blood loss, the patient remained hemodynamically compensated, with a stable pulse but developed severe hypertension (BP: 200/120 mmHg), possibly stress-related or secondary to pain and physiological response. Bilateral round ligaments, uterine arteries, and tubo-ovarian pedicles were sequentially clamped, cut, and ligated, and the uterus was removed and sent for histopathological examination. Cervical stump closure and layered abdominal closure were performed with placement of a drain in situ. Hemostasis was secured, and the procedure was completed without complications. The patient was monitored in the postoperative unit for 48 hours and had an uneventful recovery. She was discharged in stable condition on her 10th postoperative day. Suture removal was done on day 10, and the sutures were healthy. Histopathological examination reported "uterus with cast," which lacks specificity. In the absence of identifiable chorionic villi, retained products of conception were considered unlikely. The findings were interpreted as an organized intrauterine clot or decidual cast.

The ultrasonographic findings of intrauterine hematoma correlated intraoperatively with hemorrhagic collection and complete cesarean scar dehiscence. The excised intrauterine material, though reported as "uterine cast," likely represented organized blood clot or decidual tissue rather than retained products of conception.

## Discussion

Delayed PPH is an uncommon condition and is not well-studied due to its low incidence. Among patients who develop delayed PPH, only a small proportion are reported in the literature, as most cases are managed on an outpatient basis. Even in patients who present to the emergency department, diagnosis can be challenging because there is no universally accepted definition to classify delayed PPH as mild, moderate, or severe [7].

The presence of leukopenia and thrombocytopenia in this case required consideration of sepsis, systemic inflammatory response, or consumptive coagulopathy. However, negative cultures and stable coagulation parameters argued against these possibilities, suggesting a reactive or dilutional cause.

Severe secondary PPH caused by uterine scar dehiscence is extremely rare. The bleeding usually occurs due to shearing of blood vessels along the margins of the uterine scar. Known risk factors for scar dehiscence include diabetes, emergency cesarean section, infection, poor suture technique, hematoma formation along the uterine incision line, and a low-placed incision in the lower uterine segment [7,8]. Previously published case reports describing similar presentations are summarized in Table 2.

**Table 2:** Summary of previously published case reports. LSCS: lower-segment cesarean section.

Author	Day of presentation	Presenting complaint	Obstetric history	Cause of postpartum hemorrhage	Management	Postoperative outcome
Chavan et al. [9]	14 days after LSCS	Abdominal pain, fever with chills, loose stools with fecal incontinence	P2L2	Uterine infection with secondary peritoneal involvement	Hysterectomy	Uneventful; discharged on the 10th postoperative day
Thakur et al. [10]	44 days postpartum	Severe vaginal bleeding with the passage of a large clot	P2L2	Multiparity, diabetes, emergency surgery, infection, and low uterine incision	Total abdominal hysterectomy	Uneventful
Gavit and Sharma [11]	58 days after LSCS	Vaginal bleeding, giddiness, breathlessness for 5 hours	P1L1, previous LSCS, infection	Poor healing and thinning of the previous cesarean scar, resulting in secondary postpartum uterine rupture	Exploratory laparotomy	Uneventful; discharged after 13 days
Aggarwal et al. [12]	42 days postpartum	Vaginal bleeding and abdominal pain	P2L2A1	Poor scar healing combined with infection and repeated interventions, leading to cesarean scar dehiscence	Suction evacuation followed by total abdominal hysterectomy	Uneventful; discharged after 10 days

Compared to previously reported cases, our patient presented later (day 60 vs. 14-58 days) and lacked common risk factors such as infection or diabetes. While prior studies frequently implicate infection in scar dehiscence, our case demonstrated sterile cultures, suggesting hematoma-related mechanical weakening as the likely etiology.

In the present case, the patient presented on postoperative day 60 with recurrent episodes of heavy vaginal bleeding, without any history of gestational diabetes or obstetric complications. Causes of secondary PPH include retained placental fragments, endometritis, uterine subinvolution, arteriovenous malformation, gestational trophoblastic disease, and, rarely, uterine scar dehiscence [8]. Previous reports have described patients presenting on the 14th and 58th postoperative days with painless, heavy, and recurrent vaginal bleeding. Reported risk factors include previous LSCS and infections; however, in the present case, microbial culture showed no growth.

In the present case, ultrasonography of the abdomen and pelvis revealed a bulky uterus (12.4 × 6.4 × 5.4 cm) with a completely echogenic endometrial cavity. Hypoechoic areas with internal moving echoes were noted in the fundal region, suggestive of intrauterine hematoma with active bleeding, while the bilateral ovaries and adnexa appeared normal. The ultrasonographic finding of intrauterine hematoma corresponded intraoperatively to hemorrhagic collection and scar dehiscence, supporting a causal relationship between hematoma formation and scar weakening.

The uterine scar dehiscence in this patient likely occurred due to poor healing of the previous cesarean scar, compounded by hematoma formation along the incision line, which weakened the scar and predisposed it to separation, resulting in secondary postpartum hemorrhage.

In the present case, conservative management was not feasible due to profuse bleeding from an eroded uterine artery. Surgical options include scar edge revision, internal iliac artery ligation, or hysterectomy. Given the life-threatening hemorrhage requiring multiple blood and product transfusions, a hysterectomy was performed.

This case is unique due to its delayed presentation beyond six weeks, absence of infection, and association with intrauterine hematoma, which is less commonly reported as a primary contributing factor. This case highlights that the absence of infection does not exclude scar dehiscence, and clinicians should maintain a high index of suspicion in delayed postpartum bleeding.

## Conclusions

Secondary PPH due to uterine scar dehiscence is rare but can be life-threatening if not recognized promptly. Persistent bleeding, anemia, or poor response to conventional management should alert clinicians to this possibility. This case underscores the importance of considering uterine scar dehiscence in patients presenting with delayed secondary PPH, even beyond six weeks postpartum and in the absence of infection. Recognition of atypical features and timely surgical intervention are critical to preventing life-threatening complications.

As standardized guidelines for managing secondary PPH are limited, individualized care is critical. Awareness of scar dehiscence as a potential cause allows timely decision-making, helping to minimize blood loss, reduce the need for emergency hysterectomy, and improve maternal outcomes.
